# Population attributable fraction of modifiable risk factors for incident hypertension: an analysis of large-scale epidemiological cohort

**DOI:** 10.1038/s41440-026-02570-3

**Published:** 2026-03-04

**Authors:** Masachika Nishikawa, Yuta Suzuki, Hidehiro Kaneko, Akira Okada, Norifumi Takeda, Hiroyuki Morita, Katsuhito Fujiu, Tatsuhiko Azegami, Kaori Hayashi, Kaori Kitaoka, Katsuyuki Miura, Atsushi Mizuno, Akihiro Nomura, Kazuomi Kario, Koichi Node, Hideo Yasunaga, Masaomi Nangaku, Hisatomi Arima, Norihiko Takeda

**Affiliations:** 1 Department of Cardiovascular Medicine, The University of Tokyo, Tokyo, Japan; 2 Center for Outcomes Research and Economic Evaluation for Health, National Institute of Public Health, Saitama, Japan; 3 Department of Advanced Cardiology, The University of Tokyo, Tokyo, Japan; 4 Department of Prevention of Diabetes and Lifestyle-Related Diseases, Graduate School of Medicine, The University of Tokyo, Tokyo, Japan; 5 Division of Nephrology, Endocrinology and Metabolism, Department of Internal Medicine, Keio University School of Medicine, Tokyo, Japan; 6 NCD Epidemiology Research Center, Shiga University of Medical Science, Otsu, Japan; 7 Department of Public Health, Shiga University of Medical Science, Otsu, Japan; 8 Department of Cardiology, St Luke’s International Hospital, Tokyo, Japan; 9 Department of Cardiovascular Medicine, Kanazawa University Graduate School of Medical Sciences, Kanazawa, Ishikawa Japan; 10 Division of Cardiovascular Medicine, Department of Medicine, Jichi Medical University School of Medicine, Tochigi, Japan; 11 Department of Cardiovascular Medicine, Saga University, Saga, Japan; 12 Department of Clinical Epidemiology and Health Economics, School of Public Health, The University of Tokyo, Tokyo, Japan; 13 Division of Nephrology and Endocrinology, The University of Tokyo Graduate School of Medicine, Tokyo, Japan; 14 Department of Preventive Medicine and Public Health, Faculty of Medicine, Fukuoka University, Fukuoka, Japan

**Keywords:** Hypertension, Population attributable fraction, Risk factors, Obesity, Age

## Abstract

Identifying and prioritizing modifiable risk factors is crucial for the primary prevention of hypertension. However, large-scale data on the population attributable fraction (PAF) for a comprehensive range of modifiable risk factors for incident hypertension in the Japanese population have been scarce. This study analyzed 1,069,948 participants (median age 56, 43.7% men) without a history of hypertension from the DeSC database. Using Cox proportional hazards models, we evaluated the association between modifiable risk factors (obesity, diabetes mellitus, dyslipidemia, smoking, habitual alcohol consumption, physical inactivity, and sleep disorders) and incident hypertension to calculate their PAFs. Over a median follow-up of 3.64 years, 116,690 new hypertension diagnoses were recorded. Obesity had the highest PAF at 6.36%, followed by sleep disorder (4.11%), current smoking (3.39%), dyslipidemia (2.74%), habitual alcohol consumption (2.10%), physical inactivity (1.93%), and diabetes mellitus (1.55%). The PAF of obesity for incident hypertension decreased with age, from 15.10% among individuals aged <40 years to 7.93% among those aged 40–64 years and 3.70% among those aged ≥65 years. Similarly, obesity’s PAF was higher in men (7.93%) than in women (5.02%). The total PAF for all evaluated modifiable risk factors showed a more pronounced contribution among younger adults and men. In conclusion, this research reveals that obesity is the largest modifiable contributor to incident hypertension in the Japanese population. Furthermore, the impact of modifiable risk factors for hypertension is more significant in younger adults and men. These findings offer valuable insights for developing effective public health policies aimed at preventing hypertension.

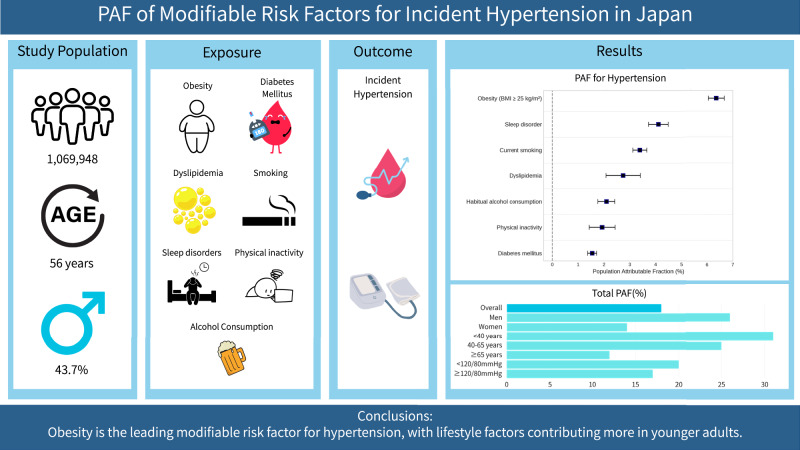

## Introduction

Hypertension is a major risk factor for cardiovascular disease (CVD) [1] and represents a significant public health challenge in Japan [2, 3]. Its development is closely linked to modifiable risk factors such as obesity, smoking, and physical inactivity, making intervention in these factors a cornerstone of preventive strategy [4–6].

Many previous epidemiological studies have identified modifiable risk factors for hypertension [7–15]. While interventions targeting these factors are widely recommended for the prevention and management of hypertension [4–6], the population-level impact of eliminating these risk factors on the incidence of hypertension has not been fully quantified.

The population attributable fraction (PAF) is an epidemiological measure commonly used to quantify the public health impact of specific exposures [16]. It measures the extent to which hypertension incidence in a population is attributable to a known risk factor [17], thus offering actionable guidance for advanced population-level approaches to hypertension prevention. Despite its importance for public health planning, no large-scale study has comprehensively estimated the PAF of multiple risk factors on the incidence of hypertension in the Japanese population. In addition, the extent to which these contributions vary by age and sex has not been examined.

The objective of this study was, therefore, to use a large-scale database primarily to calculate the PAF for multiple modifiable risk factors for incident hypertension, and to further examine how these contributions differ by age and sex.

## Methods

### Study design

This study was a retrospective longitudinal cohort study using the DeSC database, a large-scale medical information database from Japan covering the period from April 2014 to August 2023, provided by DeSC Healthcare, Inc. (Tokyo, Japan) [18–20]. This database is considered highly representative of the national population as it integrates data from Japan’s three major health insurance systems: health insurance societies for employees of large companies (Kempo), National Health Insurance for the self-employed and others (Kokuho), and the Medical Care System for the Advanced Elderly (for those aged ≥75 years). The DeSC database population exhibited an age distribution similar to national population estimates. The prevalence rates for diabetes mellitus and hypertension were comparable to those reported in the National Health and Nutrition Survey [19, 20].

The database contains two main sources of information: annual health check-up data and insurance claims data. The health check-up data includes detailed information such as anthropometric and blood pressure measurements, fasting laboratory values, medical history, and lifestyle questionnaires. These check-ups are widely promoted in Japan, with high participation rates [21]. The claims data includes comprehensive records of inpatient and outpatient diagnoses, coded according to the International Classification of Diseases, Tenth Revision (ICD-10), as well as prescription history.

### Study participants

**Fig. 1:**
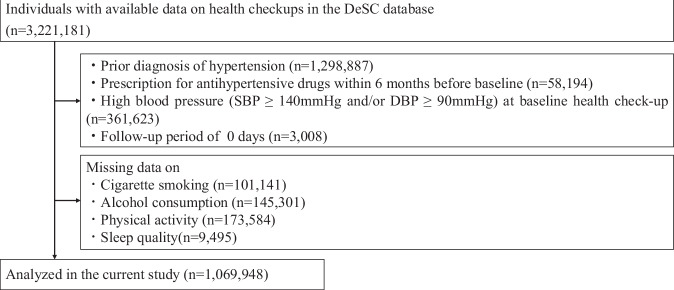
Flowchart. From a large-scale health check-up database in Japan, we identified individuals who underwent a baseline health check-up. We excluded individuals for the following reasons: a history of hypertension (*n* = 1,298,887); use of antihypertensive medication within 6 months prior to baseline (*n* = 58,194); presence of hypertension at the baseline check-up (*n* = 361,623); an insufficient follow-up period (0 days; *n* = 3008); and missing data on key lifestyle factors such as smoking status, alcohol consumption, physical activity, or sleep quality (total *n* = 429,521). After these exclusions, 1,069,948 participants were included in the final analysis

We extracted individuals from the DeSC database who had undergone a baseline health check-up. From this population, we excluded participants based on the following criteria: (1) a history of hypertension prior to the baseline survey (*n* = 1,298,887); (2) a prescription for antihypertensive drugs within 6 months before baseline (*n* = 58,194); (3) having hypertension at the time of the baseline health check-up (*n* = 361,623); and (4) a follow-up period of 0 days (*n* = 3008). Furthermore, individuals with missing data for necessary covariates were also excluded: (5) smoking status (*n* = 101,141); (6) alcohol consumption habits (*n* = 145,301); (7) physical activity habits (*n* = 173,584); and (8) sleep quality (*n* = 9495). Based on these criteria, the final study population comprised 1,069,948 individuals (Fig. 1).

### Ethical approval

The University of Tokyo’s Ethics Committee granted approval for this study (approval number: 2021010NI), which was conducted in accordance with the Declaration of Helsinki. As all data were de-identified, the requirement for informed consent was waived.

### Variables and measurement

We collected the following data using standardized procedures at each participant’s initial health check-up: Body Mass Index (BMI), blood pressure, and blood test values. Following the protocol recommended by the Japanese Ministry of Health, Labour, and Welfare, blood pressure was measured by healthcare professionals using a standard sphygmomanometer or an automated device on the right arm after participants had rested for 5 min in a seated position. The average of two measurements on a single occasion was typically used for analyses [22]. Information on cigarette smoking (current or noncurrent/never), alcohol consumption (daily or not daily), physical activity (active or inactive), and restfulness from sleep (for the assessment of sleep disorder) (good or poor) was collected from a standardized self-reported questionnaire during the health checkup. Physical inactivity was defined as not engaging in 30 min of exercise at least twice a week or walking for ≥ 1 h/day.

Obesity was defined, based on the criteria of the Japan Society for the Study of Obesity, as a BMI of ≥25 kg/m² at the baseline health check-up [23]. Diabetes was defined as a hemoglobin A1c of ≥6.5% or use of glucose-lowering medications. Dyslipidemia was defined as a low-density lipoprotein cholesterol level of ≥140 mg/dL, a high-density lipoprotein cholesterol level of < 40 mg/dL, a triglyceride level of ≥150 mg/dL, or use of lipid-lowering medications.

### Outcome

The primary outcome was new-onset (incident) hypertension during the follow-up period. Incident hypertension was identified from claims data based on International Classification of Diseases, Tenth Revision (ICD-10) codes (I10-I15).

### Statistical analysis

Continuous variables were presented as median (interquartile range), and categorical variables as number (percentage). We used multivariable Cox proportional hazards models, adjusted for age, sex, systolic blood pressure, diastolic blood pressure, and all other modifiable risk factors, to assess the association between each risk factor and incident hypertension, calculating hazard ratios (HRs) and their 95% confidence intervals (CIs). Multicollinearity among covariates included in the multivariable Cox regression models was evaluated using variance inflation factors (VIFs).

Using these adjusted HRs and the prevalence of each factor in the population, we calculated the PAF and its 95% CI for each risk factor. We estimated the PAF and corresponding 95% CI using the Stata command “punafcc” [24, 25]. The PAF was interpreted as the proportion of new-onset hypertension that could be attributed to each factor, representing the potential reduction in risk if the factor were eliminated. We also calculated the PAF for all risk factors, assuming a scenario in which all risk factors were eliminated.

Next, we calculated P values for interaction between each risk factor and age group (<40, 40–64, vs. ≥65 years), sex, and blood pressure (systolic blood pressure <120 mmHg and diastolic blood pressure <80 mmHg vs. systolic blood pressure ≥120 mmHg and/or diastolic blood pressure ≥80 mmHg). Subsequently, we stratified the participants by these background factors and conducted similar multivariable Cox proportional hazards models and PAF calculations within each subgroup.

For sensitivity analysis, we repeated the primary analysis using a stricter definition for the outcome: a diagnosis by ICD-10 code combined with a prescription for antihypertensive drugs. Subsequently, we performed multivariable Poisson models to assess the association between each risk factor and incident hypertension.

We performed complete case analyses (i.e., excluding individuals with any missing covariate data from the sample [Fig. 1]). The level of statistical significance was set at *P* < 0.05. All statistical analyses were performed using STATA v19 (StataCorp LLC, College Station, TX).

## Results

### Clinical characteristics of participants

The clinical characteristics of the study participants are summarized in Table 1. Of the 1,069,948 final participants, the median age at the baseline health check-up was 56 years (interquartile range [IQR], 44–66 years), and 43.7% (*n* = 467,137) were men. At baseline, the most prevalent risk factor was dyslipidemia at 47.0%, followed by physical inactivity at 44.1%. The prevalence of obesity was 17.9%, habitual alcohol consumption was 19.1%, current smoking was 17.6%, and diabetes mellitus was 5.1%. Supplementary Table 1 summarizes the clinical characteristics according to the development of hypertension during the follow-up period.

**Table 1:** Baseline Characteristics

	Total (*n* = 1,069,948)
Age, years	56 (44–66)
Male, *n* (%)	467,137 (43.7)
BMI, kg/m^2^	21.9 (19.9–24.1)
Obesity (BMI ≥ 25 kg/m²), n (%)	192,034 (17.9)
SBP, mmHg	118 (108–127)
DBP, mmHg	71 (64–78)
Diabetes mellitus, *n* (%)	54,940 (5.1)
Dyslipidemia, *n* (%)	503,368 (47.0)
Cigarette smoking, *n* (%)	188,828 (17.6)
Alcohol consumption, *n* (%)	204,036 (19.1)
Physical inactivity, *n* (%)	471,870 (44.1)
Sleep disorder, *n* (%)	332,233 (31.1)
Hemoglobin A1c, %	5.5 (5.3–5.7)
LDL-C, mg/dL	122 (102–144)
HDL-C, mg/dL	64 (53–76)
Triglycerides, mg/dL	85 (61–124)

Values are shown as *n* (%) or median (interquartile range)

*BMI* body mass index, *DBP* diastolic blood pressure, *HDL-C* high-density lipoprotein cholesterol, *LDL-C* low-density lipoprotein cholesterol, *SBP* systolic blood pressure

### Incidence of hypertension

Over a median follow-up of 3.64 years (IQR, 1.62–5.80 years), 116,690 incident hypertension diagnoses were recorded. The overall incidence rate of hypertension was 290.8 per 10,000 person-years. The incidence rate varied by the presence or absence of risk factors; for example, the rate was 416.0 (95% CI, 411.2–420.8) per 10,000 person-years in the obese group, whereas it was 265.0 (95% CI, 263.2–266.7) in the non-obese group (Supplementary Table 2).

### Association between risk factors and incident hypertension

The results of the multivariable Cox regression analysis showed that all evaluated modifiable risk factors were significantly associated with incident hypertension after adjusting for other factors (Table 2). The VIFs suggested no evidence of substantial multicollinearity among the covariates included in the model (mean VIF: 1.26). The unadjusted (univariable) hazard ratios for each factor are shown in Supplementary Table 3.

**Table 2:** Multivariable-Adjusted Hazard Ratios for the Association Between Risk Factors and Incident Hypertension

Variables	Adjusted HR (95% CI)
Age (per year)	1.05 (1.05–1.05)
Male	0.93 (0.92–0.95)
SBP (per mmHg)	1.03 (1.03–1.04)
DBP (per mmHg)	1.02 (1.02–1.02)
Obesity	1.35 (1.33–1.37)
Diabetes mellitus	1.22 (1.19–1.25)
Dyslipidemia	1.05 (1.04–1.06)
Cigarette smoking	1.23 (1.21–1.25)
Alcohol consumption	1.10 (1.08–1.12)
Physical inactivity	1.05 (1.04–1.06)
Sleep disorder	1.15 (1.13–1.16)

Hazard Ratios (HR) and 95% Confidence Intervals (CI) were estimated from a multivariable Cox proportional hazards model, adjusted for all other variables listed in the table

*DBP* diastolic blood pressure, *SBP* systolic blood pressure

### Differences in effects by age, sex, and blood pressure

To evaluate whether the association between risk factors and incident hypertension differed by age, sex or blood pressure, we tested for interactions (Supplementary Tables 4–6). A significant interaction was observed between age group (<40, 40–64 vs. ≥65 years) and obesity, diabetes mellitus, dyslipidemia, current smoking, habitual alcohol consumption, and physical inactivity (all *P* < 0.05). In contrast, no significant interaction was found for sleep disorders (*P* = 0.8272). A significant interaction with sex was observed for diabetes mellitus, dyslipidemia, current smoking, habitual alcohol consumption, and sleep disorders (all *P* < 0.05). A significant interaction with blood pressure was observed for obesity, diabetes mellitus, and dyslipidemia (all *P* < 0.05).

### Population attributable fraction (PAF)

**Fig. 2:**
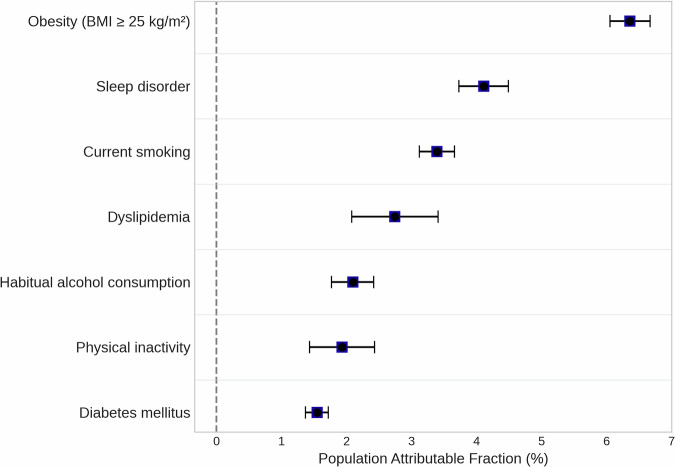
Population Attributable Fraction of Modifiable Risk Factors for Incident Hypertension in the Overall Population. The forest plot shows the Population Attributable Fraction (PAF) and its 95% Confidence Interval (CI) for each modifiable risk factor. The squares represent the point estimates of the PAF, and the horizontal lines represent the 95% CIs. Models were adjusted for age, sex, systolic blood pressure, and diastolic blood pressure

The PAF for each risk factor in the total population is shown in Fig. 2, with detailed numerical data presented in Table 3.

**Table 3:** Population Attributable Fraction (PAF) for Incident Hypertension, Overall and Stratified by Subgroups

		Age			Sex		Blood pressure	
Risk Factor	Overall	<40 years	40-64 years	≥65 years	Men	Women	Systolic blood pressure <120 mmHg and diastolic blood pressure <80 mmHg	Systolic blood pressure ≥120 mmHg and/or diastolic blood pressure ≥80 mmHg
Obesity	6.36 (6.05 to 6.67)	15.10 (12.98 to 17.16)	7.93 (7.42 to 8.43)	3.70 (3.32 to 4.08)	7.93 (7.40 to 8.45)	5.02 (4.65 to 5.38)	5.90 (5.41 to 6.38)	6.28 (5.90 to 6.67)
Diabetes mellitus	1.55 (1.37 to 1.72)	1.17 (0.69 to 1.64)	1.96 (1.73 to 2.19)	1.18 (0.90 to 1.46)	2.47 (2.16 to 2.78)	0.81 (0.61 to 1.01)	1.97 (1.66 to 2.28)	1.40 (1.19 to 1.61)
Dyslipidemia	2.74 (2.08 to 3.41)	8.99 (6.54 to 11.37)	4.46 (3.52 to 5.38)	0.07 (-0.95 to 1.09)	4.80 (3.84 to 5.75)	0.76 (-0.18 to 1.69)	3.80 (2.63 to 4.95)	1.69 (0.88 to 2.50)
Current smoking	3.39 (3.12 to 3.66)	2.87 (0.83 to 4.86)	4.44 (3.98 to 4.91)	2.06 (1.76 to 2.36)	5.49 (4.94 to 6.04)	1.53 (1.32 to 1.74)	3.47 (2.93 to 4.00)	3.25 (2.93 to 3.56)
Alcohol consumption	2.10 (1.77 to 2.42)	2.20 (0.82 to 3.56)	2.86 (2.36 to 3.36)	1.33 (0.89 to 1.77)	4.30 (3.65 to 4.95)	0.50 (0.24 to 0.77)	1.59 (1.04 to 2.13)	2.25 (1.85 to 2.64)
Physical inactivity	1.93 (1.43 to 2.43)	-4.09 (-7.57 to -0.73)	2.22 (1.40 to 3.03)	1.53 (0.92 to 2.13)	2.08 (1.32 to 2.83)	1.75 (1.09 to 2.41)	2.12 (1.15 to 3.09)	1.92 (1.35 to 2.50)
Sleep disorder	4.11 (3.73 to 4.49)	5.19 (2.70 to 7.62)	4.44 (3.83 to 5.05)	3.32 (2.85 to 3.79)	3.31 (2.75 to 3.86)	4.75 (4.23 to 5.26)	4.62 (3.85 to 5.38)	3.91 (3.48 to 4.34)
Simple Sum	22.18	31.42	28.32	13.20	30.38	15.12	23.46	20.70
All risk factors	18.49 (17.00 to 19.96)	31.39 (24.22 to 37.88)	24.60 (22.29 to 26.84)	12.22 (10.14 to 14.24)	26.37 (23.82 to 28.84)	14.45 (12.62 to 16.25)	19.96 (17.38 to 22.45)	17.27 (15.42 to 19.08)

Data are presented as Population Attributable Fraction (PAF) in percent, with the 95% Confidence Interval (CI) in brackets. Models were adjusted for age, sex, systolic blood pressure, and diastolic blood pressure. For subgroup analyses stratified by sex, sex was not included as a covariate

In the total population, the highest PAF was for obesity at 6.36% (95% CI, 6.05–6.67%), followed by sleep disorder (PAF, 4.11%), current smoking (PAF, 3.39%), dyslipidemia (PAF, 2.74%), habitual alcohol consumption (PAF, 2.10%), physical inactivity (PAF, 1.93%), and diabetes mellitus (PAF, 1.55%).

Subgroup analyses (Table 3 and Supplementary Fig. 1) revealed that these PAFs differed substantially by background factors. The PAF associated with obesity was higher in the younger and middle-aged group (<40 years and 40–64 years) at 15.10% and 7.93% compared to the older group (≥65 years) at 3.70%. The PAF for dyslipidemia was 8.99% in the <40 years group but 0.07% in the ≥65 years group (Table 3). Likewise, the PAF associated with obesity was higher in men (7.93%) than in women (5.02%).

A simple sum of the PAFs for all evaluated modifiable risk factors reached 31.42% in the <40 years and 28.32% in the 40–64 years groups, whereas it was 13.20% in the ≥65 years group. The PAFs for all evaluated modifiable risk factors were 31.39% in the <40 years and 24.60% in the 40–64 years group, whereas it was 12.22% in the ≥65 years group. Compared to women at 14.45%, the PAF for all evaluated modifiable risk factors was higher in men at 26.37%. We observed relatively similar PAFs across subgroups defined by blood pressure.

### Sensitivity analyses

To confirm the robustness of the primary findings, we conducted two sensitivity analyses. First, we changed the outcome definition to a stricter one: a diagnosis by an ICD-10 code combined with a prescription for antihypertensive drugs. Under this definition, 73,568 incident hypertension events were recorded during the follow-up period.

In this analysis, all evaluated modifiable risk factors remained significantly associated with incident hypertension. The adjusted hazard ratio for obesity was 1.40 (95% CI, 1.37–1.42). Regarding the PAF, obesity continued to have the highest contribution at 7.19% (95% CI, 6.80–7.59%), followed by current smoking (5.38%), sleep disorder (4.61%), and dyslipidemia (2.93%) (Supplementary Table 7).

Second, we performed multivariable Poisson models. All evaluated modifiable risk factors were significantly associated with incident hypertension (Supplementary Table 8).

## Discussion

This study is among the first to comprehensively calculate the PAF for multiple modifiable risk factors for incident hypertension using a large contemporary Japanese cohort of over one million individuals. The most important finding was that obesity is the largest contributing factor to incident hypertension. These findings suggest that population-wide obesity countermeasures are one of the highest priority interventions for the primary prevention of hypertension. Furthermore, this study revealed specific differences in these PAFs by age and sex. Notably, the PAF for obesity was higher in the younger and middle-aged group than in the older group, and higher in men than in women. While many previous studies have shown that the relative risk of individual risk factors is higher in younger people [26], our study is unique in quantifying these effects as population-level impact (PAF) on a large scale.

Across some observational studies in non-Japanese populations, evidence indicates that eliminating individual risk factors could prevent a proportion of new-onset hypertension cases. For example, the CRONICAS cohort study in Peru reported that the PAFs for obesity-related factors—including overweight (BMI 25 to <30 kg/m²), obesity (BMI ≥ 30 kg/m²), and central obesity—each exceeded 20% [27]. Furthermore, the Nurses’ Health Study II, which included women aged 27 to 44 years in the U.S., demonstrated that if all participants had achieved an ideal lifestyle—characterized by healthy dietary habits, regular physical activity, and normal body weight—53% (95% CI, 45–60%) of new-onset hypertension cases could have been prevented [8]. Although our findings provide evidence that interventions targeting modifiable risk factors could potentially prevent a proportion of new-onset hypertension also in the Japanese population, our estimated PAF of each risk factor, including obesity, was smaller than those reported from other countries. The relatively lower PAF observed in our study may reflect underlying differences in population characteristics, risk factor definitions, prevalence of risk factors, or methodological approaches used to estimate PAF. For instance, given that PAF is calculated based on the prevalence of a given risk factor, populations with lower prevalence of factors such as obesity (e.g., the Japanese population) are likely to demonstrate correspondingly lower PAFs for these risk factors. Additionally, some previous studies did not account for baseline blood pressure when estimating PAFs, which may have led to overestimation of the PAFs for individual risk factors.

Our findings offer three important implications for public health strategy. First, the fact that individual PAFs, and especially the PAF for all risk factors, were overwhelmingly higher in the younger and middle-aged group (~31% and 25%) compared to the older group (~12%) suggests that lifestyle factors play an extremely large role in the pathogenesis of hypertension in this younger demographic. Second, given that obesity has the largest PAF, population-wide obesity countermeasures are strongly suggested as a top-priority intervention in hypertension prevention. Third, in addition to obesity, several other modifiable risk factors, such as dyslipidemia, habitual alcohol consumption, and sleep disorders, also showed non-negligible PAFs, indicating that focusing solely on a single risk factor may be insufficient. Furthermore, in the sensitivity analysis in which hypertension was defined by combining ICD-10 diagnosis codes with antihypertensive prescriptions, we observed consistently higher PAFs across all risk factors compared to the primary analysis. Taken together, these findings highlight the need for a comprehensive, lifestyle-oriented approach that targets multiple behavioral risk factors from a young age. Such multifaceted strategies are highly likely to suppress future hypertension incidence.

Several factors may underlie the observation that PAFs for modifiable risk factors were substantially higher in the younger and middle-aged population. First, individuals who develop risk factors at a young age may represent a more “severe phenotype of risk.” This suggests a greater underlying predisposition and, importantly, leads to a longer cumulative exposure to the detrimental effects of these factors over their lifespan [28–30]. Second, from an epidemiological perspective, the relative risk associated with each factor is often mathematically amplified in younger individuals simply because their baseline absolute risk of developing hypertension is very low. In contrast, the pathophysiology of hypertension in the elderly is more complex, and the physiological aging process itself, which includes the development of age-related comorbidities and more prolonged interactions between risk factors and the environment, becomes a dominant contributor. For example, the Kailuan study has demonstrated that younger age at the onset of overweight was associated with a higher risk of hypertension compared with older age at onset [31]. Data from the Third National Health and Nutrition Examination Survey also demonstrated a stronger association between obesity and hypertension in younger and middle-aged populations compared with older populations [32]. These findings are similar to our results, suggesting that the powerful effect of aging may diminish the relative importance (and thus the PAF) of modifiable risk factors.

Furthermore, the higher PAF for factors like obesity and habitual alcohol consumption in men is likely explained by the significantly higher prevalence of these lifestyle-related risk factors in the male population, given that PAF is influenced by both relative risk and exposure prevalence [33]. In contrast, the PAF for diabetes mellitus was lower than that for other factors. However, this should not be misinterpreted as indicating that diabetes management has limited utility. Indeed, among all evaluated risk factors, individuals with diabetes exhibited the highest incidence of hypertension.

Several limitations should be considered when interpreting our results. First are limitations related to measurement. While a standardized protocol for blood pressure measurement is recommended, adherence in a nationwide real-world setting may vary, potentially introducing measurement variability. In this study, each risk factor was assessed only once at baseline, and we could not account for changes in lifestyle during the follow-up period (e.g., smoking cessation, weight change, or adoption of exercise habits). Additionally, information on risk factors such as smoking, sleep disorders, and physical inactivity was derived from self-reported data, which may introduce recall bias or underreporting. Second are limitations related to confounding. As this is an observational study, the influence of unmeasured confounding factors cannot be completely ruled out. For instance, factors such as dietary habits including salt intake and consumption of vegetables and fruit consumption (potassium intake) etc. genetic predispositions, or socioeconomic status, which were not available in this study, could potentially affect the observed associations. Therefore, while obesity had the largest contribution to hypertension among the modifiable factors evaluated in the present investigation, a re-evaluation would be necessary if factors such as salt and potassium intake were included. Third are limitations related to study design and generalizability. The PAF calculated in this study does not prove causality but is an estimate based on the strength of observed associations and exposure prevalence. Fourth, the incident hypertension was defined using ICD-10 codes. In real-world clinical practice in Japan, a hypertension diagnosis code is often recorded at the time antihypertensive medication is initiated, rather than being assigned strictly on the basis of blood pressure values measured according to guideline-based criteria. Consequently, individuals with elevated blood pressure who were managed solely with non-pharmacological interventions, such as lifestyle modification or dietary counseling, may not have been assigned an ICD-10 diagnosis code for hypertension and could have been misclassified as non-cases in the present study. Such outcome misclassification may have resulted in an under-ascertainment of true incident hypertension and could have biased the estimated associations and corresponding population attributable fractions, most plausibly toward the null. At the same time, prior validation studies using Japanese administrative claims data have demonstrated that claims-based algorithms for identifying hypertension show acceptable validity when compared with health screening–based definitions, supporting the use of ICD-10–based outcomes in large-scale epidemiological research [34]. To further address this limitation, we conducted a sensitivity analysis using a stricter outcome definition requiring both an ICD-10 diagnosis code and a prescription for antihypertensive medication, and the results were consistent with those of the primary analysis. Fifth, in this analysis, the presence of dyslipidemia and diabetes was associated with an increased likelihood of a hypertension diagnosis. Although this finding is pathophysiologically reasonable, it is also important to consider the possibility that regular hospital visits for dyslipidemia or diabetes may have contributed to a higher probability of detecting hypertension (detection bias). Sixth, in the subgroup analysis, physical inactivity was paradoxically associated with a lower risk of incident hypertension in the younger age group. This finding may be due to chance as a result of multiple comparisons, or to the possibility that the present questionnaire did not adequately capture physical activity levels among younger individuals. Further investigation is warranted to clarify this issue. Finally, a point regarding the interpretation of the results. While our findings indicate that the “population-level contribution” of modifiable risk factors is higher in younger people, the “individual absolute risk” of developing hypertension is naturally higher in the elderly. From a public health perspective, it is essential to consider both viewpoints to identify the optimal use of preventive medical resources.

In conclusion, these large-scale data reveal that among the modifiable factors evaluated, obesity is the leading risk factor contributing to incident hypertension in the Japanese population. We also found that the contribution of modifiable risk factors is greater in younger people than in the elderly, and in men than in women. To reduce the lifetime burden of hypertension, it is crucial from an epidemiological perspective to strengthen comprehensive lifestyle interventions. These interventions should particularly target younger and middle-aged populations and men, with a focus on population-wide obesity countermeasures.

## Supplementary information

Supplementary figure

Supplementary table

Supplementary information

## Data Availability

The DeSC database is available for anyone who purchases it.
