# Supplementary material for: Population attributable fraction of modifiable risk factors for incident hypertension: an analysis of large-scale epidemiological cohort
Source: Hypertens Res. 2026 Mar 4;49(5):1726–35. doi: 10.1038/s41440-026-02570-3 (PMC13148980; doi:10.1038/s41440-026-02570-3)

## Slide 1
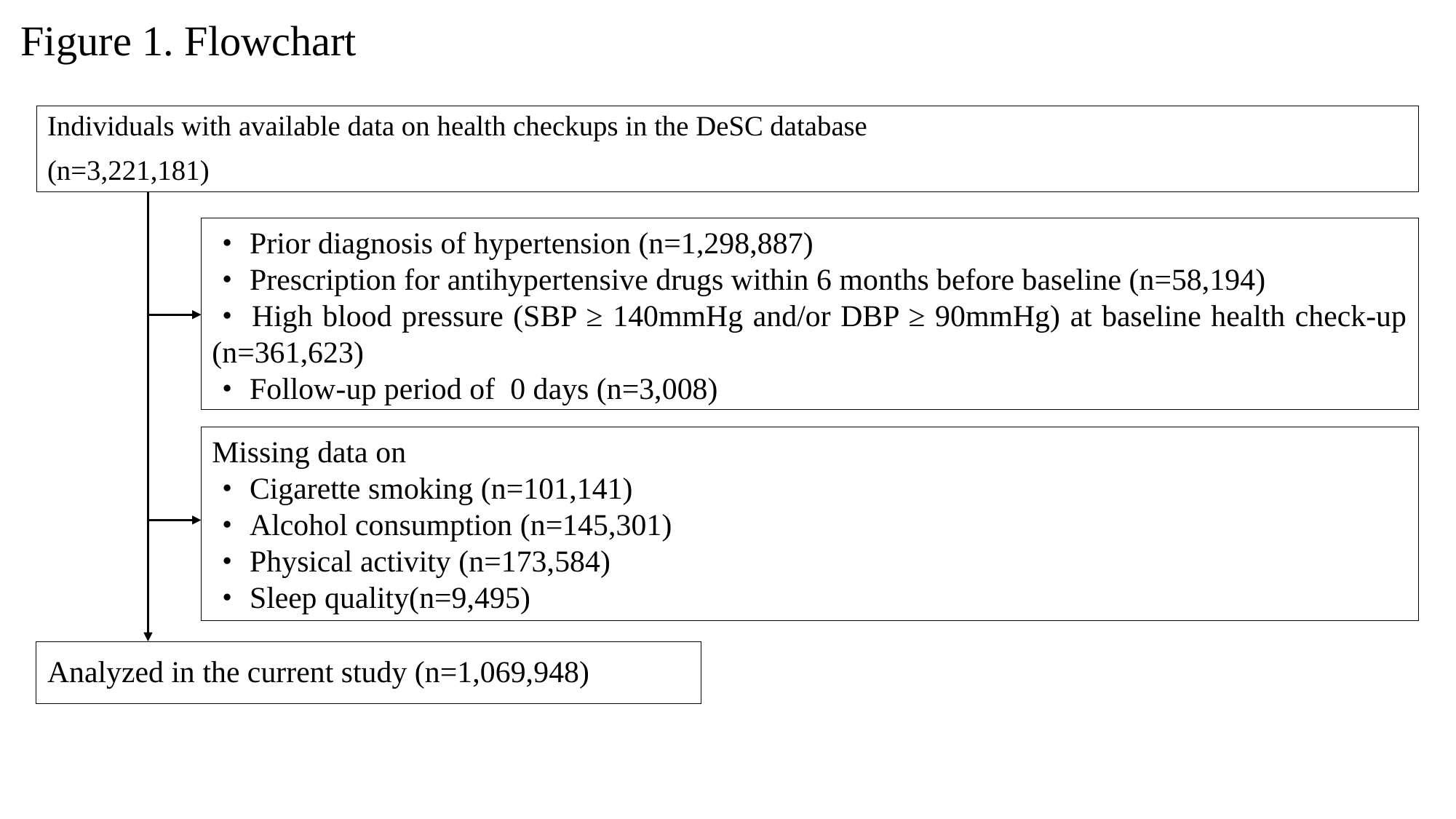

Figure 1. Flowchart
Individuals with available data on health checkups in the DeSC database
(n=3,221,181)
・Prior diagnosis of hypertension (n=1,298,887)
・Prescription for antihypertensive drugs within 6 months before baseline (n=58,194)
・High blood pressure (SBP ≥ 140mmHg and/or DBP ≥ 90mmHg) at baseline health check-up (n=361,623)
・Follow-up period of 0 days (n=3,008)
Missing data on
・Cigarette smoking (n=101,141)
・Alcohol consumption (n=145,301)
・Physical activity (n=173,584)
・Sleep quality(n=9,495)
Analyzed in the current study (n=1,069,948)

## Slide 2
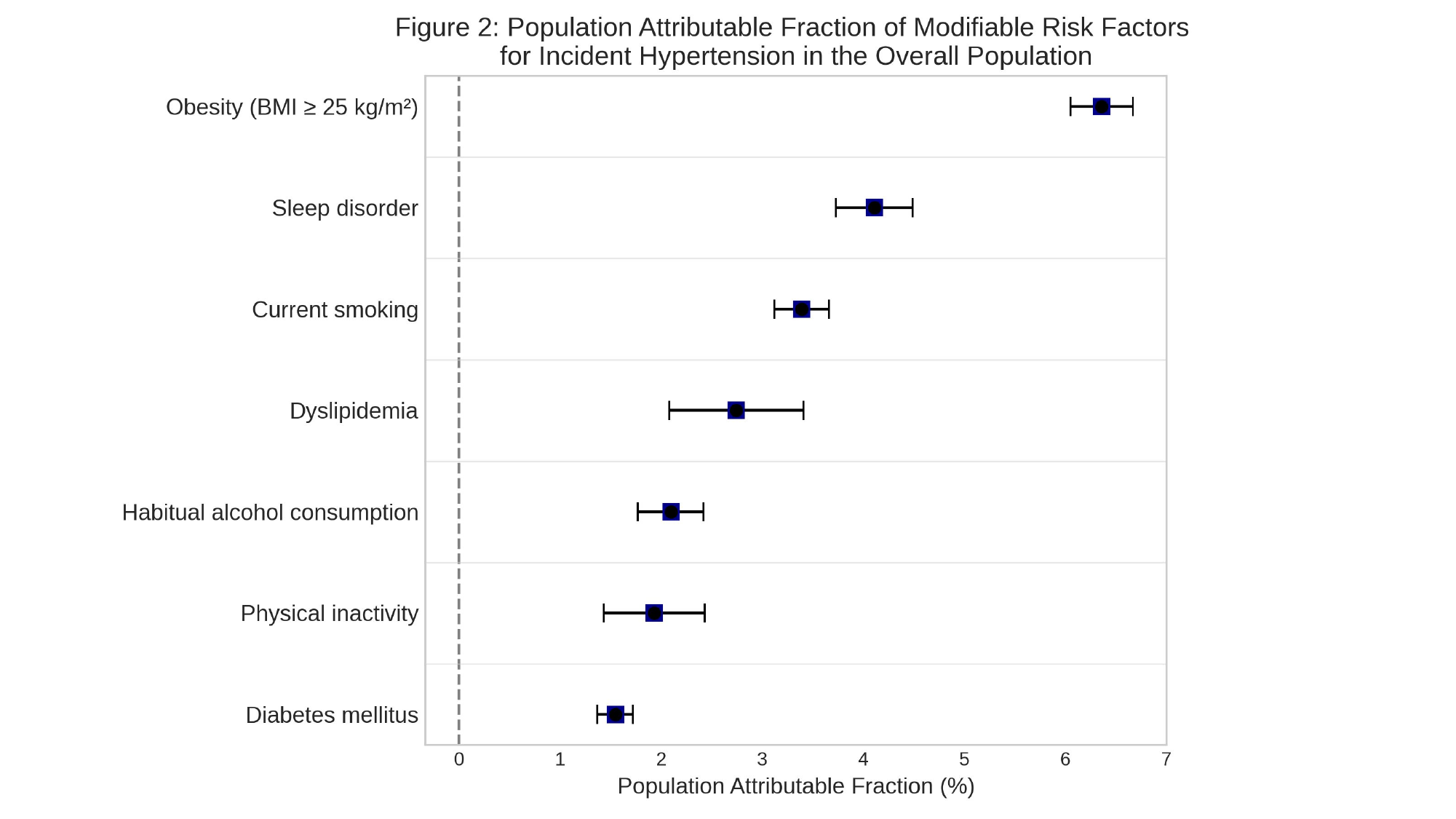

## Slide 3
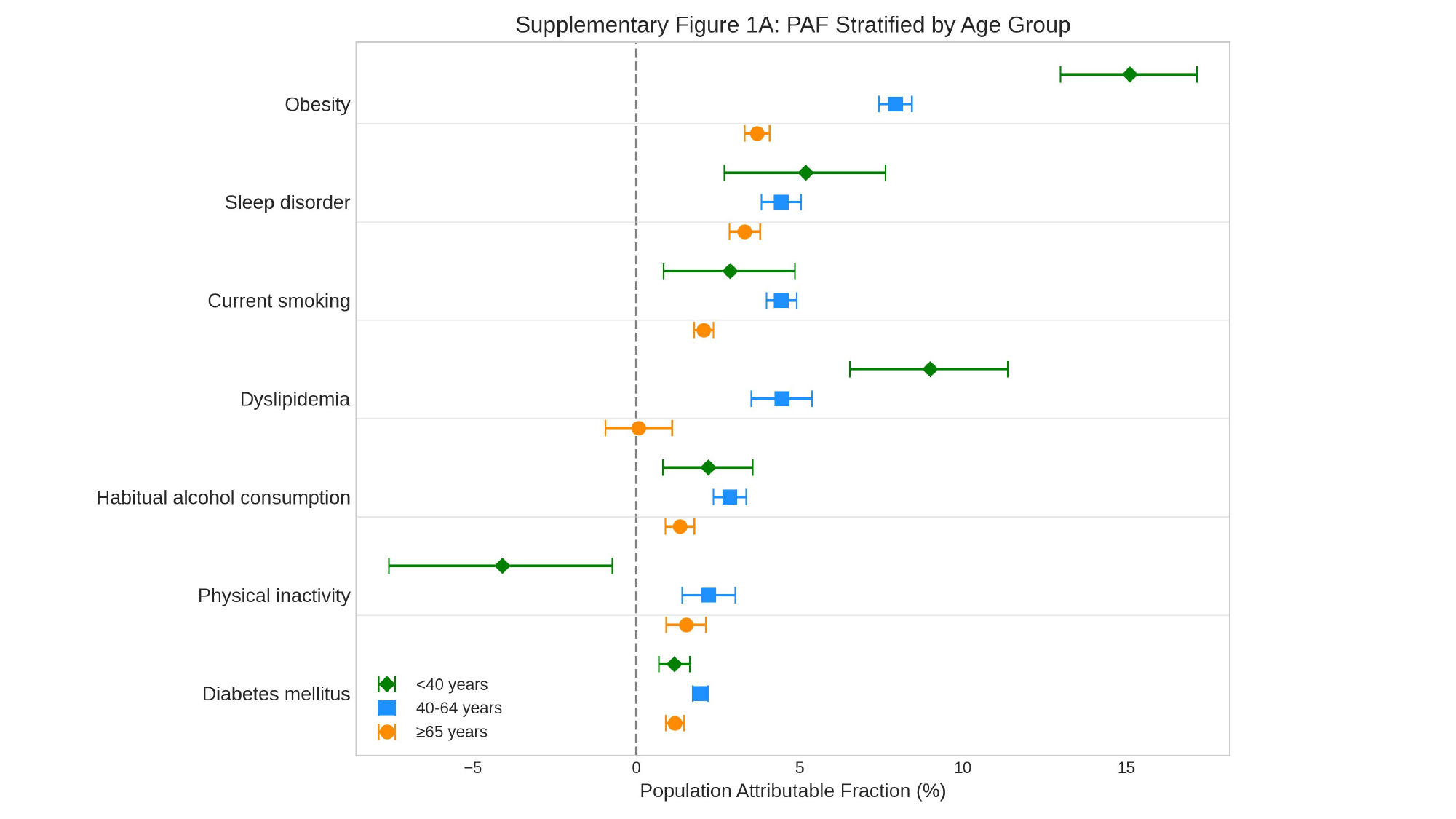

## Slide 4
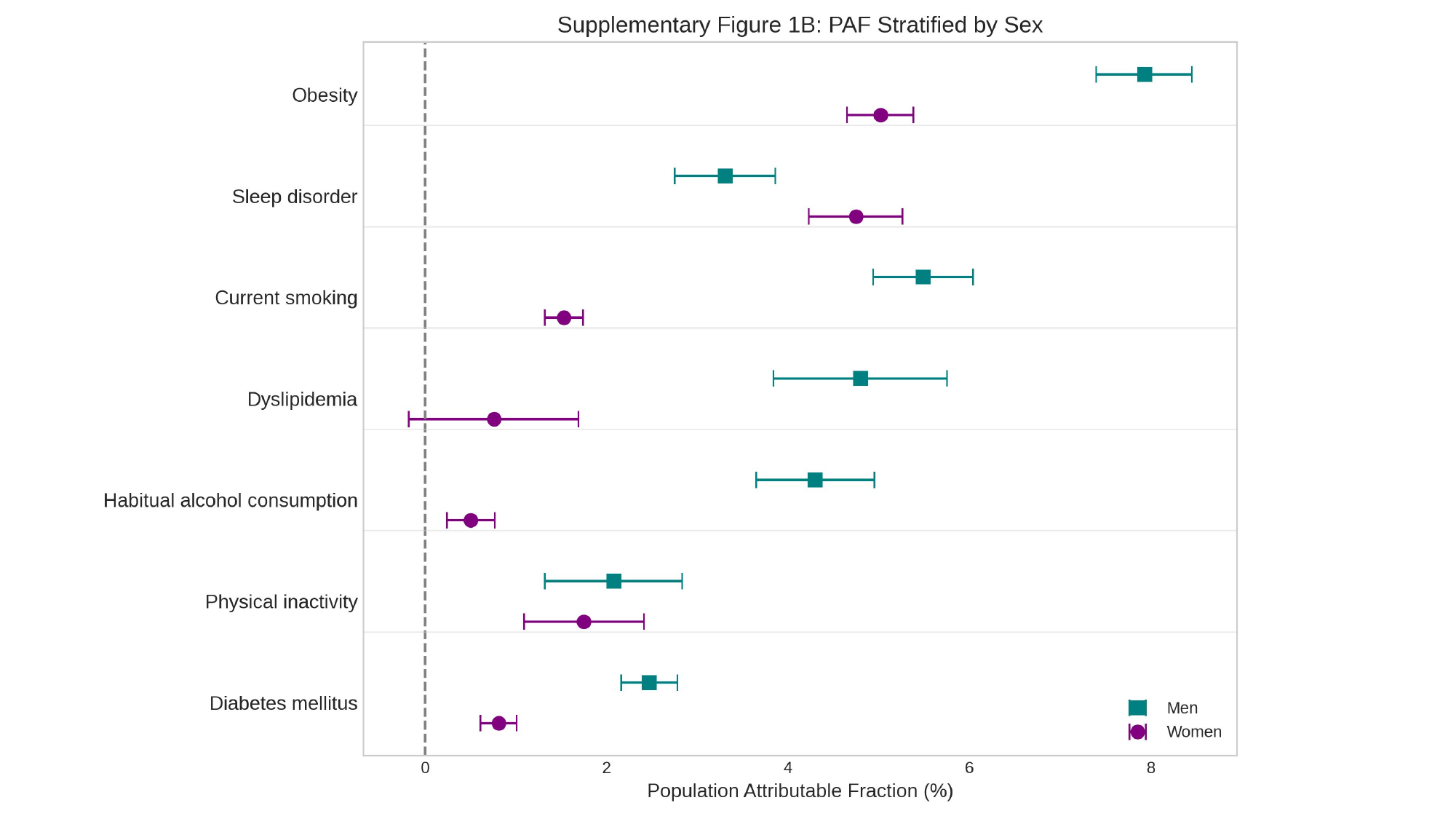

## Slide 5
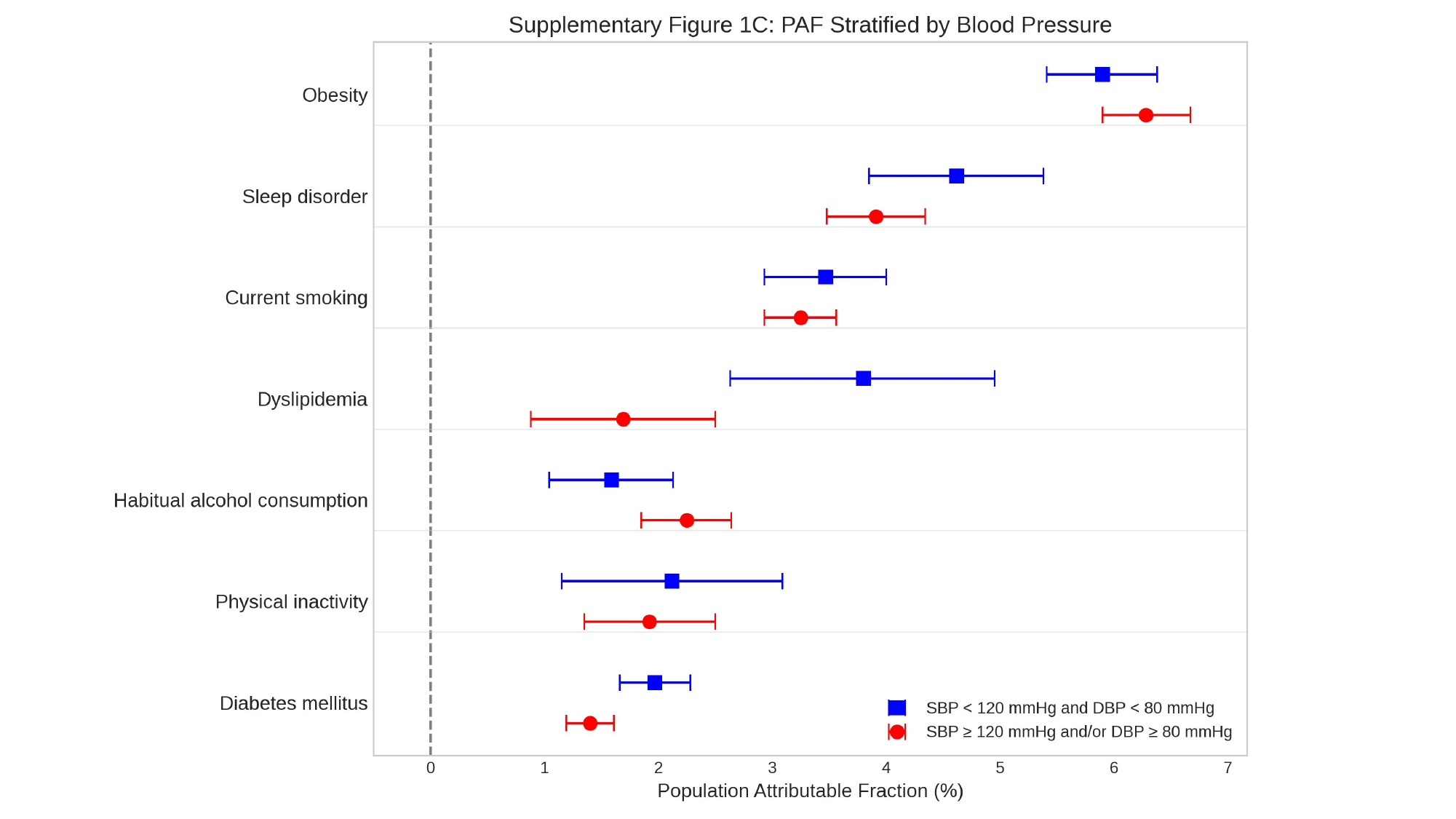

Supplement: Supplementary file 1 — Supplementary figure [file 41440_2026_2570_MOESM1_ESM.pptx]
