# Supplementary material for: Population attributable fraction of modifiable risk factors for incident hypertension: an analysis of large-scale epidemiological cohort
Source: Hypertens Res. 2026 Mar 4;49(5):1726–35. doi: 10.1038/s41440-026-02570-3 (PMC13148980; doi:10.1038/s41440-026-02570-3)
Supplement: Supplementary file 2 — Supplementary table [file 41440_2026_2570_MOESM2_ESM.docx]

**Supplementary Table 1.** **Baseline Characteristics Stratified by the Development of Hypertension during the Follow-up Period**

|  | Incidence of Hypertension (-) (n= 953,258) | Incidence of Hypertension (+) (n=116,690) |
| --- | --- | --- |
| Age, years | 55 (43-66) | 64 (54-68) |
| Male, n (%) | 414,279 (43.5) | 52,858 (45.3) |
| BMI, kg/m^2^ | 21.8 (19.8-24.0) | 22.7 (20.6-24.9) |
| Obesity (BMI ≥ 25 kg/m²), n (%) | 163,507 (17.2) | 28,527 (24.4) |
| SBP, mmHg | 117 (108-126) | 126 (118-132) |
| DBP, mmHg | 70 (64-77) | 75 (69-81) |
| Diabetes mellitus, n (%) | 44,909 (4.7) | 10,031 (8.6) |
| Dyslipidemia, n (%) | 436,976 (45.8) | 66,392 (56.9) |
| Cigarette smoking, n (%) | 167,673 (17.6) | 21,155 (18.1) |
| Alcohol consumption, n (%) | 177,213 (18.6) | 26,823 (23.0) |
| Physical inactivity, n (%) | 421,936 (44.3) | 49,934 (42.8) |
| Sleep disorder, n (%) | 295,295 (31.0) | 36,938 (31.7) |
| Hemoglobin A1c, % | 5.5 (5.3-5.7) | 5.6 (5.4-5.9) |
| LDL-C, mg/dL | 122 (102-144) | 126 (106-147) |
| HDL-C, mg/dL | 64 (53-77) | 61 (51-74) |
| Triglycerides, mg/dL | 84 (60-122) | 96 (69-138) |

Values are shown as n (%) or median (interquartile range).

BMI, body mass index; DBP, diastolic blood pressure; HDL-C, high-density lipoprotein cholesterol; LDL-C, low-density lipoprotein cholesterol; SBP, systolic blood pressure.

**Supplementary Table 2. Incidence Rates of Hypertension, Stratified by Risk Factor Status**

| Risk Factor | Category | No. of Participants | Person-Years | No. of Events | Incidence Rate (per 10,000 person-years) (95% CI) |
| --- | --- | --- | --- | --- | --- |
| Obesity | No | 877,914 | 3,327,447 | 88,163 | 264.96 (263.21-266.71) |
|  | Yes | 192,034 | 685,814 | 28,527 | 415.96 (411.16-420.81) |
| Diabetes mellitus | No | 1,015,008 | 3,834,669 | 106,659 | 278.14 (276.48-279.82) |
|  | Yes | 54,940 | 178,592 | 10,031 | 561.67 (550.79-572.77) |
| Dyslipidemia | No | 566,580 | 2,197,420 | 50,298 | 228.90 (226.90-230.90) |
|  | Yes | 503,368 | 1,815,841 | 66,392 | 365.63 (362.86-368.42) |
| Current smoking | No | 881,120 | 3,279,240 | 95,535 | 291.33 (289.49-293.19) |
|  | Yes | 188,828 | 734,021 | 21,155 | 288.21 (284.35-292.12) |
| Alcohol consumption | No | 865,912 | 3,245,178 | 89,867 | 276.92 (275.12-278.74) |
|  | Yes | 204,036 | 768,083 | 26,823 | 349.22 (345.07-353.42) |
| Physical inactivity | No | 598,078 | 2,190,504 | 66,756 | 304.75 (302.45-307.07) |
|  | Yes | 471,870 | 1,822,757 | 49,934 | 273.95 (271.56-276.36) |
| Sleep disorder | No | 737,715 | 2,708,696 | 79,752 | 294.43 (292.39-296.48) |
|  | Yes | 332,233 | 1,304,565 | 36,938 | 283.14 (280.27-286.05) |

Incidence rates of hypertension stratified by baseline risk factor status. Rates are presented per 10,000 person-years with their 95% Confidence Intervals (CI).

**Supplementary Table 3. Univariable Hazard Ratios for the Association Between Risk Factors and Incident Hypertension**

| Variables | HR (95% CI) |
| --- | --- |
| Age (per year) | 1.05 (1.05-1.05) |
| Male | 0.97 (0.96-0.99) |
| SBP (per mmHg) | 1.06 (1.06-1.06) |
| DBP (per mmHg) | 1.06 (1.05-1.06) |
| Obesity | 1.57 (1.55-1.60) |
| Diabetes mellitus | 2.03 (1.99-2.08) |
| Dyslipidemia | 1.60 (1.58-1.62) |
| Cigarette smoking | 0.99 (0.97-1.00) |
| Alcohol consumption | 1.26 (1.24-1.28) |
| Physical inactivity | 0.90 (0.89-0.91) |
| Sleep disorder | 0.96 (0.95-0.97) |

Univariable hazard ratios (HR) and 95% Confidence Interval (95% CI) for the association between each baseline risk factor and incident hypertension.

**Supplementary Table 4. Interaction Between Risk Factors and Age on the Risk of Incident Hypertension**

|  | Age |  |  |  |
| --- | --- | --- | --- | --- |
|  | <40 years | 40-64 years | ≥65 years |  |
| Risk Factor | HR (95% CI) | HR (95% CI) | HR (95% CI) | P-value for Interaction |
| Obesity | 1.70 (1.59-1.83) | 1.37 (1.35-1.40) | 1.24 (1.21-1.27) | <0.001 |
| Diabetes mellitus | 1.96 (1.59-2.42) | 1.38 (1.34-1.43) | 1.13 (1.10-1.16) | <0.001 |
| Dyslipidemia | 1.30 (1.21-1.40) | 1.09 (1.07-1.11) | 1.00 (0.98-1.02) | <0.001 |
| Current smoking | 1.11 (1.03-1.19) | 1.23 (1.20-1.25) | 1.21 (1.18-1.25) | 0.03 |
| Alcohol consumption | 1.16 (1.06-1.26) | 1.12 (1.10-1.15) | 1.07 (1.05-1.09) | 0.003 |
| Physical inactivity | 0.93 (0.87-0.99) | 1.05 (1.03-1.06) | 1.04 (1.03-1.06) | 0.001 |
| Sleep disorder | 1.15 (1.07-1.22) | 1.14 (1.12-1.16) | 1.15 (1.12-1.17) | 0.83 |

Hazard Ratios (HR) and 95% Confidence Intervals (CI) were estimated from a multivariable Cox proportional hazards model. P-values are for the interaction term between each risk factor and the age category in the multivariable Cox proportional hazards model. Models were adjusted for age, sex, systolic blood pressure, and diastolic blood pressure.

**Supplementary Table 5. Interaction Between Risk Factors and Sex on the Risk of Incident Hypertension**

|  | Sex |  |  |
| --- | --- | --- | --- |
|  | Men | Women |  |
| Risk Factor | HR (95% CI) | HR (95% CI) | P-value for Interaction |
| Obesity | 1.35 (1.33-1.38) | 1.35 (1.32-1.37) | 0.73 |
| Diabetes mellitus | 1.27 (1.23-1.30) | 1.16 (1.12-1.20) | <0.001 |
| Dyslipidemia | 1.09 (1.07-1.11) | 1.01 (1.00-1.03) | <0.001 |
| Current smoking | 1.21 (1.19-1.24) | 1.27 (1.23-1.31) | 0.01 |
| Alcohol consumption | 1.13 (1.11-1.15) | 1.05 (1.02-1.08) | <0.001 |
| Physical inactivity | 1.05 (1.03-1.07) | 1.04 (1.03-1.06) | 0.63 |
| Sleep disorder | 1.12 (1.10-1.14) | 1.17 (1.15-1.19) | <0.001 |

Hazard Ratios (HR) and 95% Confidence Intervals (CI) were estimated from a multivariable Cox proportional hazards model. P-values are for the interaction term between each risk factor and the sex category in the multivariable Cox proportional hazards model. Models were adjusted for age, systolic blood pressure, and diastolic blood pressure.

**Supplementary Table 6. Interaction Between Risk Factors and Blood Pressure on the Risk of Incident Hypertension**

|  | Blood pressure |  |  |
| --- | --- | --- | --- |
|  | Systolic blood pressure <120 mmHg and diastolic blood pressure <80 mmHg | Systolic blood pressure ≥120 mmHg and/or diastolic blood pressure ≥80 mmHg |  |
| Risk Factor | HR (95% CI) | HR (95% CI) | P-value for Interaction |
| Obesity | 1.51 (1.47-1.56) | 1.30 (1.28-1.32) | <0.001 |
| Diabetes mellitus | 1.37 (1.31-1.43) | 1.18 (1.16-1.21) | <0.001 |
| Dyslipidemia | 1.08 (1.05-1.10) | 1.03 (1.02-1.04) | <0.001 |
| Current smoking | 1.23 (1.19-1.27) | 1.22 (1.20-1.24) | 0.64 |
| Alcohol consumption | 1.09 (1.06-1.13) | 1.10 (1.08-1.12) | 0.67 |
| Physical inactivity | 1.05 (1.03-1.08) | 1.05 (1.03-1.06) | 0.78 |
| Sleep disorder | 1.16 (1.13-1.18) | 1.15 (1.13-1.16) | 0.50 |

Hazard Ratios (HR) and 95% Confidence Intervals (CI) were estimated from a multivariable Cox proportional hazards model. P-values are for the interaction term between each risk factor and the sex category in the multivariable Cox proportional hazards model. Models were adjusted for age, sex, systolic blood pressure, and diastolic blood pressure.

**Supplementary Table 7. Results of Sensitivity Analysis Using a Stricter Definition of Hypertension**

| Risk Factor | Adjusted Hazard Ratio [95% CI] | Population Attributable Fraction [%, 95% CI] |
| --- | --- | --- |
| Obesity | 1.40 (1.37-1.42) | 7.19 (6.80 to 7.59) |
| Diabetes mellitus | 1.30 (1.26-1.33) | 2.18 (1.95 to 2.42) |
| Dyslipidemia | 1.05 (1.04-1.07) | 2.93 (2.08 to 3.78) |
| Current smoking | 1.38 (1.35-1.41) | 5.38 (5.03 to 5.73) |
| Alcohol consumption | 1.12 (1.10-1.14) | 2.48 (2.07 to 2.90) |
| Physical inactivity | 1.07 (1.05-1.09) | 2.79 (2.17 to 3.41) |
| Sleep disorder | 1.17 (1.15-1.19) | 4.61 (4.14 to 5.08) |

Results of the sensitivity analysis where incident hypertension was defined more strictly as a diagnosis with an International Classification of Diseases, Tenth Revision (ICD-10) code combined with a prescription for an antihypertensive drug. PAF, Population Attributable Fraction; CI, Confidence Interval. Models were adjusted for age, sex, systolic blood pressure, and diastolic blood pressure.

**Supplementary Table 8. Multivariable-Adjusted Incident Rate Ratios for the Association Between Risk Factors and Incident Hypertension**

| Variables | Adjusted IRR (95% CI) |
| --- | --- |
| Age (per year) | 1.04 (1.04-1.05) |
| Male | 0.94 (0.93-0.95) |
| SBP (per mmHg) | 1.03 (1.03-1.03) |
| DBP (per mmHg) | 1.02 (1.02-1.02) |
| Obesity | 1.34 (1.32-1.36) |
| Diabetes mellitus | 1.21 (1.19-1.24) |
| Dyslipidemia | 1.05 (1.04-1.07) |
| Cigarette smoking | 1.22 (1.20-1.24) |
| Alcohol consumption | 1.10 (1.09-1.12) |
| Physical inactivity | 1.05 (1.04-1.06) |
| Sleep disorder | 1.15 (1.14-1.17) |

Incident Rate Ratios (IRR) and 95% Confidence Intervals (CI) were estimated from a multivariable Poisson model, adjusted for all other variables listed in the table.

DBP, diastolic blood pressure; SBP, systolic blood pressure.
