# Supplementary material for: Population attributable fraction of modifiable risk factors for incident hypertension: an analysis of large-scale epidemiological cohort
Source: Hypertens Res. 2026 Mar 4;49(5):1726–35. doi: 10.1038/s41440-026-02570-3 (PMC13148980; doi:10.1038/s41440-026-02570-3)
Supplement: Supplementary file 3 — Supplementary information [file 41440_2026_2570_MOESM3_ESM.docx]

**Supplementary Figure 1. Population Attributable Fraction of Modifiable Risk Factors for Incident Hypertension, Stratified by Subgroups.**

(A) Forest plot showing the Population Attributable Fraction (PAF) and its 95% Confidence Interval (CI) for each risk factor, stratified by age group (<40, 40–65 vs. ≥65 years).

(B) Forest plot showing the PAF and its 95% CI for each risk factor, stratified by sex.

(C) Forest plot showing the PAF and its 95% CI for each risk factor, stratified by blood pressure (systolic blood pressure <120 mmHg and diastolic blood pressure <80 mmHg vs. systolic blood pressure ≥120 mmHg and/or diastolic blood pressure ≥80 mmHg).

The squares/circles represent the point estimates of the PAF, and the horizontal lines represent the 95% CIs.
